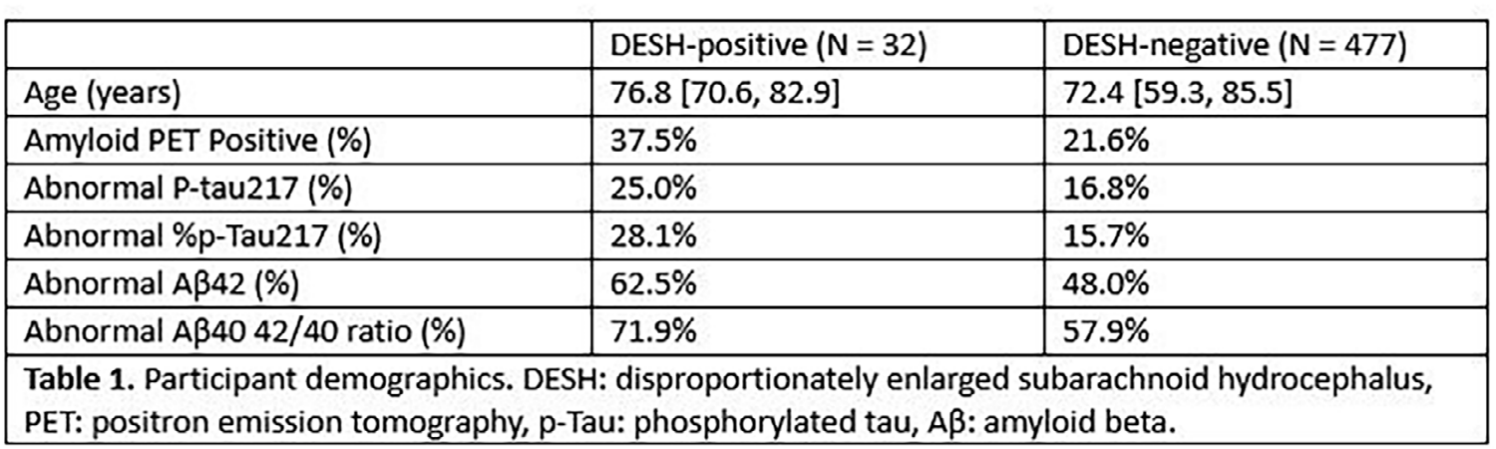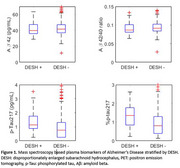# Plasma Alzheimer's mass spectrometry biomarkers associated with cerebrospinal fluid dynamics

**DOI:** 10.1002/alz70856_101248

**Published:** 2025-12-24

**Authors:** Camilo Bermudez, Ekaterina I. Hofrenning, Jeffrey L. Gunter, Petrice M Cogswell, David T. Jones, Christopher G Schwarz, Val J Lowe, Benjamin D Elder, Ronald Petersen, Prashanthi Vemuri, Alicia Algeciras‐Schimnich, Michelle M Mielke, David S. Knopman, Neill R. Graff‐Radford, Clifford R. Jack, Jonathan Graff‐Radford

**Affiliations:** ^1^ Mayo Clinic, Rochester, MN, USA; ^2^ Department of Radiology, Mayo Clinic, Rochester, MN, USA; ^3^ Department of Neurology, Mayo Clinic, Rochester, MN, USA; ^4^ Division of Public Health Sciences, Wake Forest University, School of Medicine, Winston‐Salem, NC, USA; ^5^ Mayo Clinic in Florida, Jacksonville, FL, USA

## Abstract

**Background:**

Individuals with coexisting Alzheimer's disease (AD) and disorders of CSF dynamics such as normal‐pressure hydrocephalus (NPH) have lower CSF Aβ42 levels, which can confound interpretation of AD CSF‐biomarkers. However, little is known about how CSF dynamics influences plasma AD biomarkers. The objective of this study was to determine whether mass spectrometry‐based blood tests for AD are associated with disproportionately enlarged subarachnoid space hydrocephalus (DESH) on MRI.

**Method:**

509 Individuals from the Mayo Clinic Study of Aging, with MRI, 11C‐Pittsburgh compound B (Aβ) PET scans, and plasma phosphorylated‐tau protein 217, Aβ40 and Aβ42 were included. We performed an age‐adjusted logistic regression to determine whether abnormal values of *p*‐Tau 217 (Abnormal if >1.55 pg/mL), %p‐tau217 defined as the ratio of phosphorylated Tau217 to non‐phosphorylated tau x 100% (Abnormal if >1.55%), Aβ42 (Abnormal if <41.5 pg/mL), and Aβ42/40 ratio (Abnormal if <0.094) were associated with the presence of DESH, as classified by a previously validated automated algorithm.

**Result:**

Thirty‐two participants were classified as DESH‐positive. The median [IQR] age was 76.8 yrs [70.6, 82.9] for participants with DESH and 72.4 [59.3, 85.5] without DESH (Table 1). An abnormal plasma Aβ42 was associated with DESH (OR, 2.56 [1.19, 5.74], *p* = 0.018), but having an abnormal Aβ42/40 ratio (OR 2.01 [0.94, 4.56], *p* = 0.079) was not. There was no association between being DESH‐positive and an abnormal plasma *p*‐tau217 (OR 1.30 [0.51, 3.09], *p* = 0.560) or with abnormal %plasma *p*‐tau217 (OR 1.82 [0.74, 4.20], *p* = 0.17) (Figure 1). Subgroup analysis showed that only those participants who had an abnormal Aβ42 and positive amyloid PET (≥25 Centiloids) showed an association with DESH compared to biomarker‐ and PET‐negative participants (OR 3.10 [1.15, 8.43], *p* = 0.024*)*.

**Conclusion:**

Understanding how CSF dynamics affect plasma biomarkers is critical for implementation. The relationship between lower Aβ42 and DESH is expected based on prior CSF studies, which can be addressed by using an amyloid ratio. *p*‐tau217 shows robustness against DESH, making it a suitable biomarker in cases of abnormal cerebrospinal fluid dynamics although validation in a more severe CSF dynamics group is warranted.